# Alterations in lipid metabolism of spinal cord linked to amyotrophic lateral sclerosis

**DOI:** 10.1038/s41598-019-48059-7

**Published:** 2019-08-12

**Authors:** Adriano Britto Chaves-Filho, Isabella Fernanda Dantas Pinto, Lucas Souza Dantas, Andre Machado Xavier, Alex Inague, Rodrigo Lucas Faria, Marisa H. G. Medeiros, Isaias Glezer, Marcos Yukio Yoshinaga, Sayuri Miyamoto

**Affiliations:** 1 Departamento de Bioquímica, Instituto de Química, Universidade de São Paulo, São Paulo, Brazil; 2 Departamento de Bioquímica, Escola Paulista de Medicina, Universidade Federal de São Paulo, São Paulo, Brazil

**Keywords:** Amyotrophic lateral sclerosis, Lipidomics

## Abstract

Amyotrophic lateral sclerosis (ALS) is characterized by progressive loss of upper and lower motor neurons leading to muscle paralysis and death. While a link between dysregulated lipid metabolism and ALS has been proposed, lipidome alterations involved in disease progression are still understudied. Using a rodent model of ALS overexpressing mutant human Cu/Zn-superoxide dismutase gene (SOD1-G93A), we performed a comparative lipidomic analysis in motor cortex and spinal cord tissues of SOD1-G93A and WT rats at asymptomatic (~70 days) and symptomatic stages (~120 days). Interestingly, lipidome alterations in motor cortex were mostly related to age than ALS. In contrast, drastic changes were observed in spinal cord of SOD1-G93A 120d group, including decreased levels of cardiolipin and a 6-fold increase in several cholesteryl esters linked to polyunsaturated fatty acids. Consistent with previous studies, our findings suggest abnormal mitochondria in motor neurons and lipid droplets accumulation in aberrant astrocytes. Although the mechanism leading to cholesteryl esters accumulation remains to be established, we postulate a hypothetical model based on neuroprotection of polyunsaturated fatty acids into lipid droplets in response to increased oxidative stress. Implicated in the pathology of other neurodegenerative diseases, cholesteryl esters appear as attractive targets for further investigations.

## Introduction

The central nervous system (CNS) is characterized by the presence of high amounts and a wide variety of lipids^1^. According to their molecular characteristics and cellular localization, lipids play a critical role in the CNS controlling membrane fluidity^2^, improving transmissions of electrical signals and serving as precursors for various second messengers^1^. Although molecular alterations in CNS at different stages of life are part of the physiological development^3^, aging can modulate alterations in the lipidome, especially in response to increased reactive oxygen species (ROS)^4,5^. As a consequence, alterations in lipid metabolism could contribute to the onset of neurodegenerative disorders^6^.

This paper is focused on ALS, a neurodegenerative disorder characterized by loss of motor neurons in CNS. Symptomatic stages of ALS lead to muscular atrophy, paralysis and death within 5 years after the onset of symptoms^7,8^. Among familial cases of ALS, G93A mutation in the gene that encodes for the antioxidant enzyme Cu/Zn-superoxide dismutase (SOD1-G93A) is one of the most studied models^8^. The putative mechanisms involved in ALS progression consist in protein aggregation of mutant SOD1, abnormal production of ROS and alterations in mitochondrial functions^8–10^. Lipid alterations in ALS have been mainly investigated through targeted analysis of some specific lipid species. Historically, a pioneer study by Cutler *et al*. (2002) revealed increased levels of sphingolipids and cholesteryl esters based on targeted lipid analysis of the spinal cord from an ALS mouse model^11^. In addition, these authors suggested a link between sphingolipid metabolism and synthesis of cholesteryl esters in the CNS^12^. Alterations in sphingolipid metabolism were also emphasized in skeletal muscle and spinal cord from SOD1 mice^13–15^. Increased sphingolipid content, specially ceramides and glucosylceramides, as well as accumulation of phosphatidylcholine (PC 36:4) were reported as the main lipid alterations in cerebrospinal fluid of ALS patients^16^. Collectively, these studies and others have found evidence for significant lipid metabolism alterations in advanced stages of ALS^17,18^.

Here, we sought to capture global lipidome alterations linked to ALS progression by performing an untargeted mass spectrometry-based lipidomic analysis of motor cortex and spinal cord tissues from ALS asymptomatic (SOD1-G93A 70 days old) and symptomatic (SOD1-G93A 120 days old) rats in comparison to their wild types as controls. Our analysis revealed drastic changes in lipid metabolism in the CNS both according to aging and disease progression.

## Results

To investigate how lipids are affected in ALS, we performed a global lipidome analysis of motor cortex and spinal cord from asymptomatic (SOD1-G93A 70 days) and symptomatic (SOD1-G93A 120 days) ALS rats, and their respective age-matched wild type (WT 70 days and WT 120 days) as control groups. Results from these analyses are presented separately below.

### Sphingolipids are modulated according to age and slightly with disease progression in motor cortex of SOD1-G93A and WT rats

**Figure 1:**
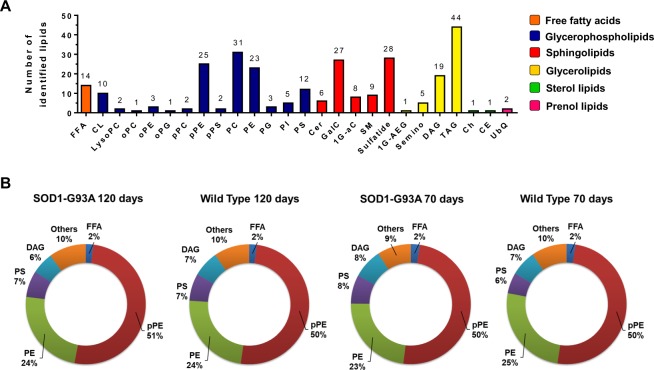
Lipidome profile in motor cortex of SOD1-G93A and WT groups at 70 and 120 days old. (**A**) Number of the identified lipid molecular species per lipid subclass. (**B**) Relative abundance of summed concentrations of each lipid subclass. Abbreviations: FFA, free fatty acids; CL, cardiolipin; PC, phosphatidylcholine; PE, phosphatidylethanolamine; PG, phosphatidylglycerol; PI, phosphatidylinositol; PS, phosphatidylserine; Cer, ceramide; GalC, galactosylceramide; 1G-aC, monoglycosylated acylceramide; SM, sphingomyelin; 1G-AEG, monoglucosyl-acyl-ether-glycerol; DAG, diacylglycerol; TAG, triacylglycerol; Ch, cholesterol; CE, cholesteryl ester; UbQ, ubiquinone; p- or o- before phospholipids (PC, PE, PG and PS) indicate plasmenyl or plasmanyl, respectively.

Using untargeted lipidomics, we identified and quantified 285 lipid molecular species in the motor cortex, which were sorted into 26 lipid subclasses (Fig. 1A). Glycerophospholipids (n = 120) followed by sphingolipids (n = 78), glycerolipids (n = 69) and free fatty acids (n = 14) showed the highest diversity of individual lipid molecular species. In terms of relative abundance, plasmenyl and diacyl phosphatidylethanolamine (pPE and PE) encompassed together about 70% in mass of the total identified lipids in motor cortex of both SOD1-G93A and WT rats (Fig. 1B). The remaining 30% of lipids were mainly composed of phosphatidylserine (PS, ~7%), diacylglycerol (DAG, ~5%), free fatty acids (FFA, ~2%), among others.

**Figure 2:**
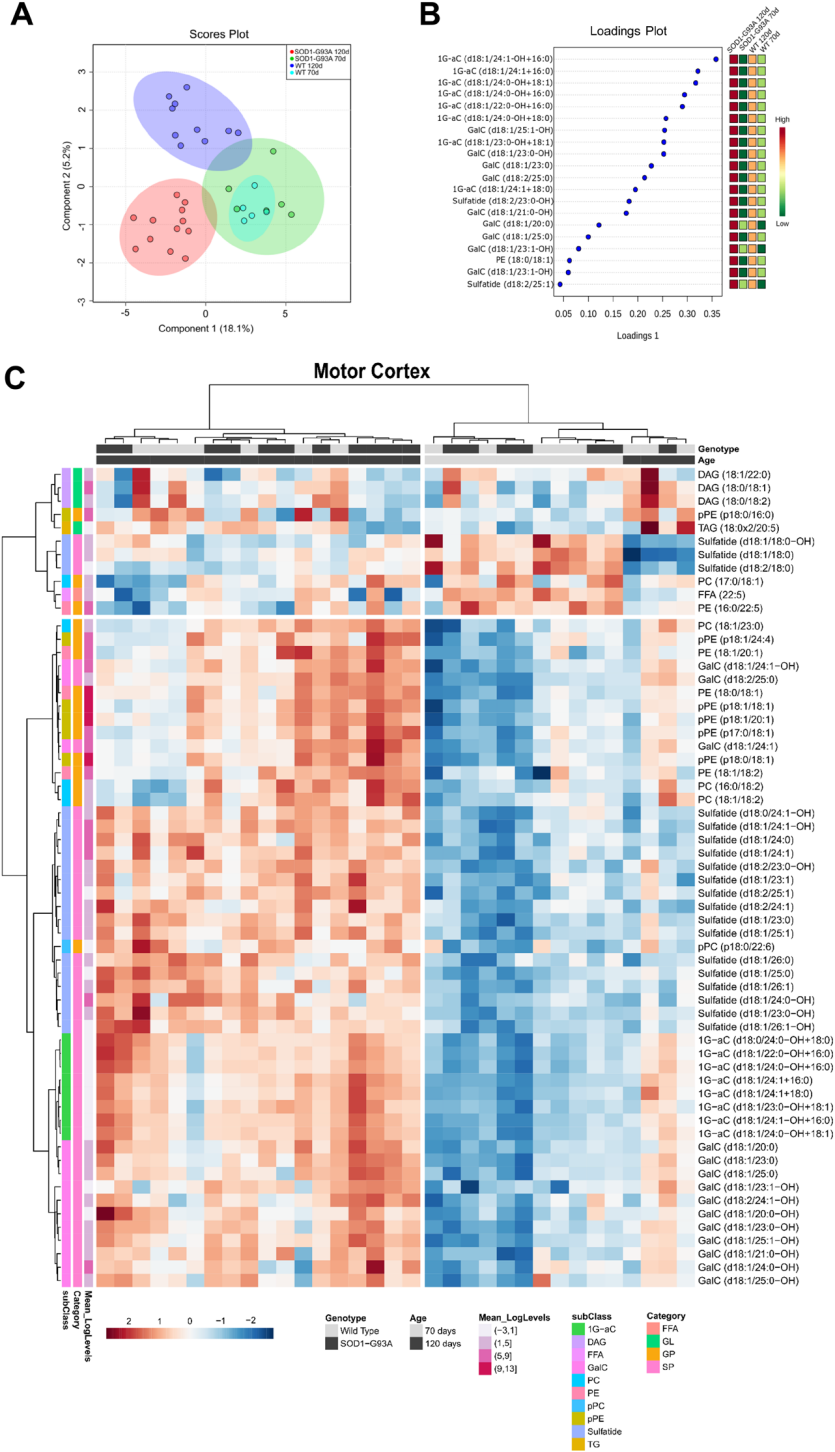
Multivariate analysis and heatmap plot of motor cortex lipidomes from SOD1-G93A and WT groups. Log transformation of lipid concentrations was applied to lipid concentrations prior to statistical analysis in Metaboanalyst. (**A**) Score plot of the sparse partial least squares analysis (sPLS-DA); (**B**) Top 20 lipid species according to loadings 1 values of the sPLS-DA (**C**) Heatmap plot displaying clusters of samples and the 61 significantly altered lipid molecular species according to one-way ANOVA followed by Tukey’s post-test (p < 0.05; FDR-adjusted). Individual lipids are shown in rows and samples displayed in columns, according to cluster analysis (clustering distance was calculated by Pearson and clustering algorithm estimated by Ward). Each colored cell on the heatmap plot corresponds to values above (red) or below (blue) the mean normalized concentrations for a given lipid. Abbreviations of lipid subclasses are described in Fig. 1. Nomenclature: “p” before a given fatty acyl chain indicates sn-1 plasmenyl followed by a sn-2 linked fatty acid (as in pPE (p18:0/18:1); “d” refers to the sphingoid base followed by a n-acyl chain, for example, Sulfatide (d18:1/24:1). “OH” refers to the hydroxyl group in the n-acyl chain, for example, Cer (18:0/24:1-OH). Please note that fatty acid composition of phospholipids, TAG and DAG does not reflect their specific position in the glycerol backbone. GL, glycerolipids; GP, glycerophospholipids; SP, sphingolipids and TG, triglycerides.

Multivariate analysis was performed by sparse partial least squares analysis (sPLS-DA) in the motor cortex lipidome. The score plot of the sPLS-DA showed a relatively clear segregation of experimental groups according to age (component 1 = 18.1%, Fig. 2A). This trend was related to high levels of several sphingolipids observed in older compared to younger animals as revealed by the top 20 loadings 1 values (Fig. 2B). Univariate analysis performed by one-way ANOVA yielded 61 altered lipid molecular species when comparing all groups. These altered lipids are displayed as clusters in the heatmap plot (Fig. 2C). Of note, most samples from both SOD1-G93A 120d and WT 120d clustered together displaying increased levels of galactosyl ceramides (GalC; 14 species), sulfatides (16 species) and monoglycosylated acylceramides (1G-aC; 8 species). The clustering of samples in the heatmap plot and sPLS-DA suggest that age, rather than the disease per se, modulates major lipidome alterations in motor cortex.

Alterations in sphingolipids concentration evidenced by both multivariate and univariate analyses occurred in several individual molecular species and included variations in the composition of sphingosine base, hydroxylation and length of n-acyl chains (Fig. S1). Taken together, our findings suggest that age plays a critical role modulating sphingolipids metabolism in motor cortex.

**Figure 3:**
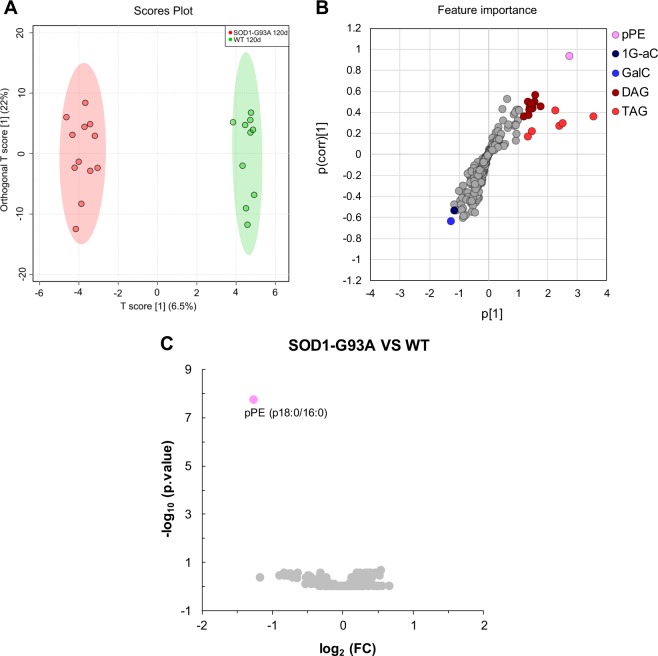
Pairwise comparison by orthogonal partial least squares–discriminate analysis (OPLS-DA) and volcano plot of motor cortex lipidomes of SOD1-G93A 120d and WT 120d groups. Data used for this comparison have been Log-transformed prior to statistical analysis in Metaboanalyst. (**A**) Score plot of the OPLS-DA revealing a clear segregation of groups; (**B**) Loadings plot of the OPLS-DA. Colored features representing lipid subclasses displaying the top 20 lipid species according to highest p[1] values; (**C**) Volcano plot of the pairwise comparison SOD1-G93A 120d versus WT 120d groups represented by the log_2_ (fold change) plotted against the –log_10_ (p value). This analysis revealed only one molecular specimen altered between groups. Statistical significance was evaluated by t-test (p < 0.05; FDR-adjusted) and the fold change was set to >1.5.

In line with previous studies^13–15^, our data indeed revealed that some sphingolipids, particularly the summed concentrations of GalC and 1G-aC (Fig. S2), were significantly increased in the SOD1-G93A 120d group relative to the other groups. This finding suggests a link between ALS progression and lipidome alterations in motor cortex, and we thus decided to examine specific differences between SOD1-G93A 120d and WT 120d groups. For this purpose, we performed a comparison between these groups through an OPLS-DA and volcano plot (Fig. 3). As shown in Fig. 3A,B, the discrimination between these groups in multivariate analysis is mainly linked to alterations in the levels of TAG, DAG and one specimen of pPE (top 20 lipid species ranked according to loading 1 values of the OPLS-DA). Differences in lipidome alterations evidenced by multivariate analysis need to be interpreted with caution since univariate analysis by volcano plot yielded a single altered specimen (i.e., pPE (p18:0/16:0)). These results reinforce the idea that lipid alterations in motor cortex are clearly more linked to age than disease progression per se.

### Ceramides, cholesteryl esters and cardiolipin are altered in spinal cord of SOD1-G93A rats

**Figure 4:**
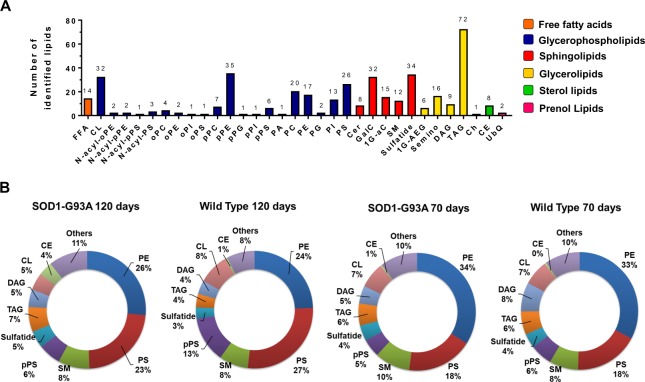
Lipidome profile identified in spinal cord of SOD1-G93A and WT groups at 70 and 120 days old. (**A**) Number of lipid species identified in each lipid subclass. (**B**) Relative abundance of summed concentrations of lipid subclasses. Abbreviations: FFA, free fatty acids; CL, cardiolipin; PA, phosphatidic acid; PC, phosphatidylcholine; PE, phosphatidylethanolamine; PG, phosphatidylglycerol; PI, phosphatidylinositol; PS, phosphatidylserine; Cer, ceramide; GalC, galactosylceramide; 1G-aC, monoglycosylated acylceramide; SM, sphingomyelin; 1G-AEG, monoglucosyl-acyl-ether-glycerol; DAG, diacylglycerol; TAG, triacylglycerol; Ch, cholesterol; CE, cholesteryl ester; UbQ, ubiquinone; p- or o- before lipid subclass mean plasmenyl or plasmanyl, respectively.

The lipidomic analysis of spinal cord revealed 406 lipid molecular species sorted into 33 lipid subclasses. Glycerophospholipids (n = 177) followed by sphingolipids (n = 101) and glycerolipids (n = 103) showed the highest diversity of individual lipid molecular species in spinal cord tissues (Fig. 4A). The most abundant lipid subclasses were PE and PS which represented together approximately 50% in mass of total lipid molecular species (Fig. 4B). Sphingolipids representing ~13% were the third most abundant lipid subclass, which showed sphingomyelins (SM) and sulfatides as the most abundant subclasses. In comparison to motor cortex lipidomes (Fig. 1), higher percentages of storage lipids (represented by triacylglycerols and cholesteryl esters) and cardiolipin (CL) were found in spinal cord tissues.

**Figure 5:**
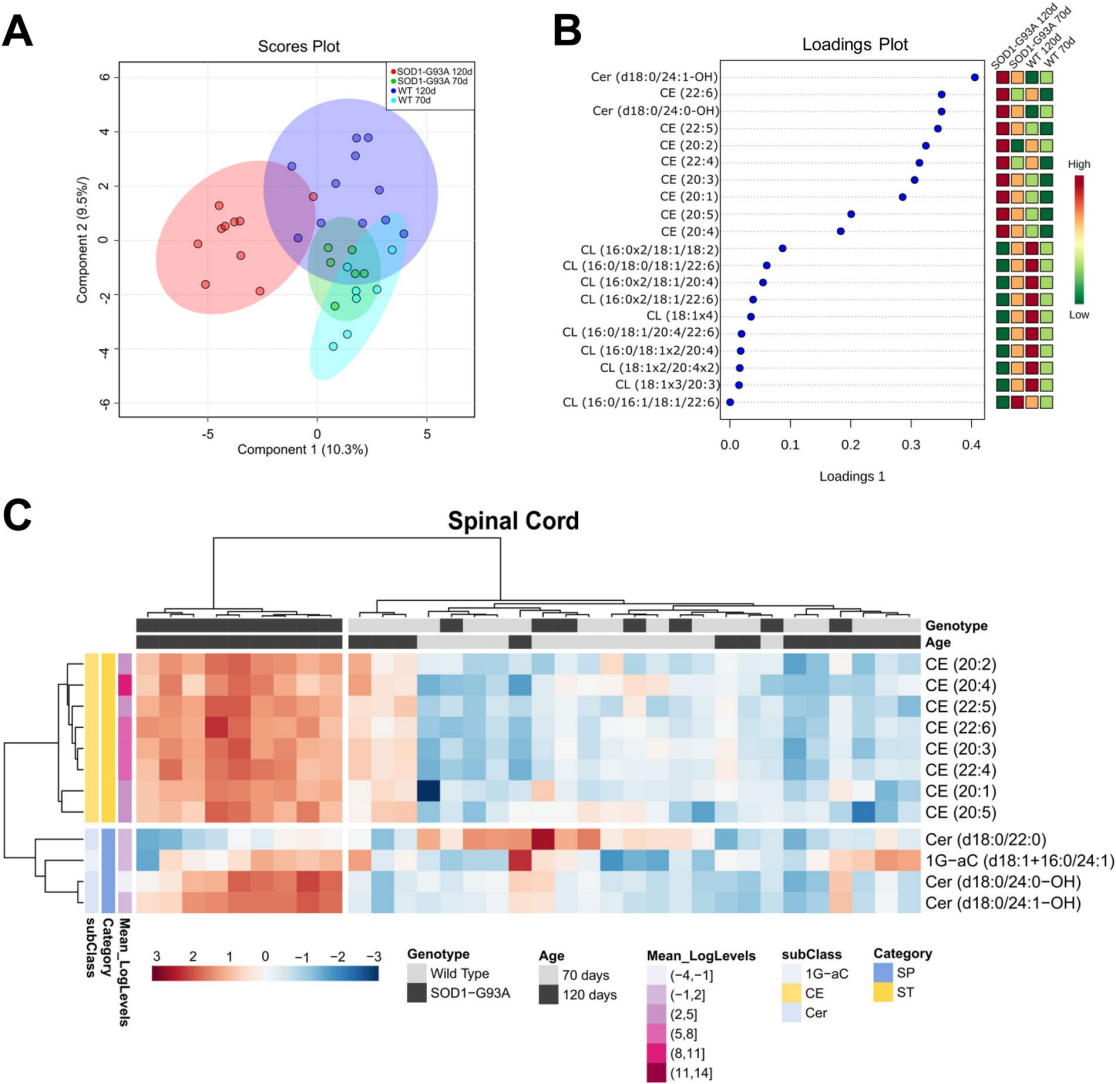
Multivariate analysis and heatmap plot of spinal cord lipidomes of SOD1-G93A and WT groups. (**A**) Score plot of the sPLS-DA; (**B**) Top 20 lipid species according to loadings 1 values of the sPLS-DA; (**C**) Heatmap plot displaying clusters of 12 significantly altered lipid molecular species (according to one-way ANOVA) and samples. For statistical parameters and graphic details see Fig. 2 caption. Abbreviations of lipid subclasses are described in Fig. 4. Nomenclature: “d” refers to the sphingoid base followed by a n-acyl chain, for example, Cer (d18:0/22:0). “OH” refers to the hydroxyl group in the n-acyl chain, for example, Cer (18:0/24:1-OH).

As shown in Fig. 5A, the spinal cord lipidome of SOD1-G93A 120d group is spatially segregated from the others as explained by component 1 of the sPLS-DA (10.3%). Analysis of the top 20 lipid species ranked according to values of loadings 1 of the sPLS-DA reveals enrichment of ceramides and cholesteryl esters in SOD1-G93A 120d group and reduced amounts of cardiolipins relative to the other groups (Fig. 5B). A clear separation of the SOD1-G93A 120d group from others is also observed in the heatmap plot (Fig. 5C), displaying the top 12 significantly altered lipid species from the spinal cord (one-way ANOVA). Univariate analysis revealed modulation of 4 sphingolipids (ceramides and 1G-aC) and all cholesteryl esters species identified by lipidomic analysis. Importantly, 2 ceramides and all cholesteryl esters were increased exclusively in the symptomatic group (Fig. 5C), linking these lipids to ALS progression.

**Figure 6:**
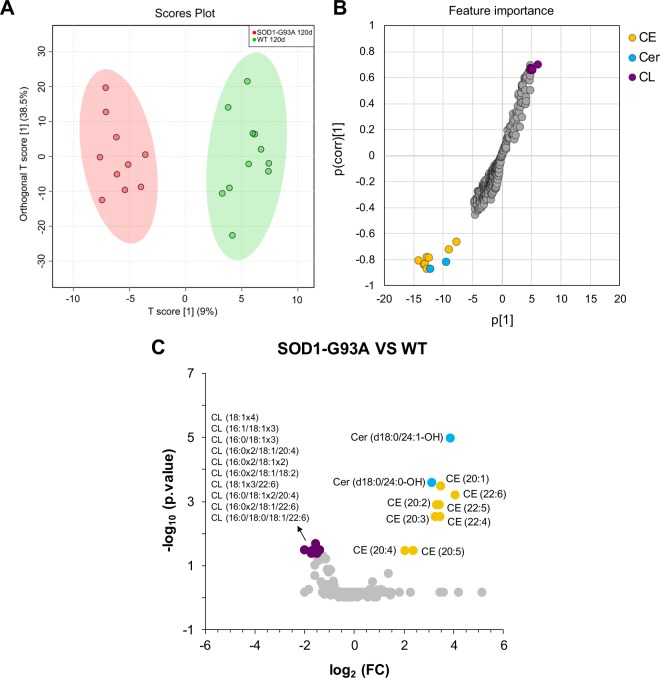
Pairwise comparison by OPLS-DA and volcano plot of spinal cord lipidomes of SOD1-G93A 120d and WT 120d groups. (**A**) Multivariate statistical analysis using orthogonal partial least squares–discriminate analysis (OPLS-DA); (**B**) Feature importance of the top 20 lipid species altered according to the OPLS-DA analysis; (**C**) Volcano plot is represented by the log_2_ (fold change) of SOD1-G93A 120d/WT 120d groups plotted against the –log_10_ (p). Statistical significance was evaluated by t-test (p < 0.05; FDR-adjusted) and the fold change was set to >1.5. The significantly altered lipid molecular species are colored according to lipid subclasses. List of altered cardiolipins is sorted by log_2_ (fold change).

To examine detailed differences linked to symptomatic stage of ALS, we performed a pairwise comparison between SOD1-G93A 120d and WT 120d groups through OPLS-DA and volcano plot (Fig. 6). Together, multivariate and univariate analyses confirmed significant alterations in ceramides and cholesteryl esters, which were both found markedly elevated in SOD1-G93A 120d group. In addition, several cardiolipins (CL) species were found reduced in the SOD1-G93A 120d group.

Among ceramide species, remarkable changes were observed in Cer (d18:0/24:0-OH) and Cer (d18:0/24:1-OH). These hydroxylated ceramides were drastically elevated in the SOD1-G93A 120d group compared to the age-matched WT group (Fig. S3). In addition, changes in ceramides levels were examined by calculating the ratios of hydroxylated/non-hydroxylated (OH/n-OH) and very long chain/long chain (VLC/LC) species. Both ratios were found significantly increased in the SOD1-G93A 120d group, whereas the ratio ceramide/dihydroceramide (Cer/dh-Cer) was reduced (Fig. S4B). Therefore, these data confirm previous findings indicating an association between ceramide metabolism/remodeling and ALS disease development^11,14,15^.

**Figure 7:**
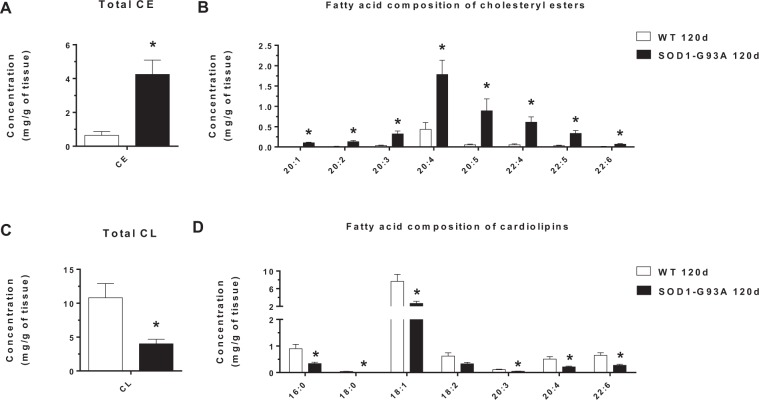
Altered concentrations of cholesteryl esters (CE) and cardiolipins (CL) identified in the spinal cord of SOD1-G93A 120d and WT 120d groups. (**A**) Concentration of total CE. (**B**) Fatty acid composition of CE. (**C**) Concentration of total CL. (**D**) Fatty acid composition of CL. Data are shown as mean ± standard error of mean (SEM). Statistical significance was evaluated by t-test (p < 0.05; FDR-adjusted) using Metaboanalyst. (*) Statistically different when compared to the WT 120d group.

The most conspicuous changes in the lipidome of spinal cord in the SOD1-G93A 120d group were related to alterations in cholesteryl esters and cardiolipin levels (Fig. 7). Of note, the SOD1-G93A 120d group showed a 6-fold increase in total concentration of cholesteryl esters (Fig. 7A). Elevated cholesteryl esters levels were mainly linked to those molecular species esterified to polyunsaturated fatty acids (PUFAs), with arachidonic (20:4), eicosapentaenoic (20:5) and adrenic (22:4) acids (Fig. 7B). Contrasting with the increase in cholesteryl esters, 10 out of 32 cardiolipin species were found reduced in the SOD1-G93A 120d group relative to the age-matched WT (Fig. S5). This change was reflected not only in total concentration of cardiolipins, which was lower in SOD1-G93A 120d group (Fig. 7C), but also in the major fatty acids esterified to cardiolipins (Fig. 7D).

Guided by findings that astrocytes isolated from the spinal cord of an identical ALS rat model has aberrant features and accumulates lipid droplets^19^, we performed immunohistochemistry of the astrocyte-specific glial fibrillary acidic protein (GFAP). This analysis was conducted to confirm astrogliosis, a hallmark of ALS, and it was restricted to the spinal cords of SOD1-G93A 120d group and its age-matched control (WT 120d). As depicted in the Fig. S6, the spinal cord of the SOD1-G93A 120d group exhibited severe astrogliosis (stellate appearance) in the grey matter of the spinal cord ventral horn, as reported in previous studies^20,21^.

## Discussion

Lipids play a critical role in structuring the CNS through membrane fluidity control, transmission of electrical signals and stabilization of synapses^2,22^. Alterations of lipid metabolism in neurons and glial cells (astrocytes, oligodendrocytes and microglia) modulate processes linked to aging and neurodegenerative diseases^23,24^. To address lipidome alterations in the CNS, we investigated the motor cortex and spinal cord of SOD1-G93A transgenic rats as model for ALS and compared them to wild type as control. In this study, an untargeted lipidomic analysis using high resolution UHPLC-Q-TOF-MS was performed. Manual identification of the most abundant molecular ions based exclusively on their MS/MS profile allowed us to unambiguously annotate a wide range of lipid molecular species occurring in motor cortex (285 species, Fig. 1) and spinal cord (406 species, Fig. 4) tissues. Major lipidome changes in the motor cortex were linked to altered sphingolipid metabolism and to animal age (Figs 2 and 3). In contrast, lipidome alterations in the spinal cord were strongly associated with ALS at symptomatic stage (SOD1-G93A 120d group) (Figs 5 and 6).

Alterations in the lipidome of motor cortex were mostly related to age, except for a single lipid (pPE (p18:0/16:0)) that was significantly decreased in SOD1-G93A 120d relative to age-matched WT (Fig. 3C). Among sphingolipids, several GalC, sulfatide and 1G-aC were increased in both WT and SOD1-G93A groups at 120d compared to 70d (Figs 2 and S1). Our findings are in line with active production of myelin by adults, either from newly formed oligodendrocytes or synthesis of new membranes by existing cells, reflecting the constant increase in sphingolipids content with age^25^. Interestingly, increased content of 1G-aC was observed in motor cortex of older animals (Fig. S2). To our knowledge, 1G-aC have never been reported in the CNS and their modulation with age together with increased GalC and sulfatide concentrations suggest a possible link of these sphingolipids to myelination and brain development.

In contrast to motor cortex, drastic differences in lipidome profiles were observed in the spinal cord of SOD1-G93A 120d rats, particularly when compared to its age-matched control (WT 120d) (Fig. 6). Major alterations were linked to cholesteryl esters and cardiolipin levels and to a minor extent in the abundance of ceramides (Figs 7 and S3). Differences in ceramide metabolism have been repeatedly reported in spinal cord tissues of ALS patients and rodent models^11,13–16^. Here, we detected increased concentrations of two hydroxylated ceramides, the Cer (d18:0/24:0-OH) and Cer (d18:0/24:1-OH). Hydroxylated ceramides are essential for myelin stability and maturation, as demonstrated by knockdown and mutations in fatty acid 2-hydroxylase (FA2H, the enzyme responsible for hydroxylation of n-acyl chains) that results in neural impairments in mice^26^ and humans^27^. Ceramides have also been implicated in cellular signaling^28^, and in ALS they are thought to activate the oxysterol-binding protein (OSBP)^11^. According to these authors, OSBP binds and delivers oxysterols to the endoplasmic reticulum thereby activating acylcoenzyme A:cholesterol acyltransferase (ACAT) yielding cholesteryl esters as products. Accumulation of either hydroxylated ceramides or dihydroceramides could also trigger cell survival events such as autophagy^27,29^. Since one of the hallmarks of ALS is the presence of cytoplasmic inclusions or protein aggregates in affected motor neurons^8,30^, the stimulation of autophagic process by these ceramides could be a potential pathway for elimination of protein aggregates and damaged organelles.

As highlighted by our data, ALS symptomatic rats displayed conspicuously elevated levels of cholesteryl esters and decreased concentrations of cardiolipin species in the spinal cord as major lipid signatures of the disease (Fig. 6). Changes in cholesteryl ester metabolism were not reflected in alterations in the pools of free cholesterol (Fig. S7) or 24-hydroxycholesterol, the latter only observed in trace amounts in spinal cord tissues (data not shown). That is, it appears that cholesterol synthesis is upregulated but does not result in accumulation of free cholesterol nor 24-hydroxycholesterol, which is generally more soluble and can cross the brain-blood-barrier at a much faster rate than cholesterol^31^.

A growing body of evidence implicates impaired energy metabolism in ALS patients and models^10^. Of note, ROS generation, protein aggregation and mitochondrial dysfunction in motor neurons represent a clinical hallmark of ALS^32–35^. Whether elevated ROS is caused by SOD1 aggregation and glutamate accumulation leading to imbalanced calcium homeostasis in neurons remains unanswered. Nonetheless, elevated ROS is associated with mitochondrial dysfunction, leading to changes that range from decreased energy metabolism to major morphological modifications. Abnormal mitochondrial morphology has been repeatedly observed in motor neurons of diverse familial cases of ALS models^36–40^. These alterations consist of mitochondria clusters, swollen mitochondria, loss of cristae and vacuoles derived from degenerating mitochondria. Reflecting dysfunctional mitochondria, our data reveal a significant decrease in cardiolipin levels in spinal cord of SOD1-G93A 120d group when compared to the WT 120d group (Fig. 6). Cardiolipin is a major phospholipid of mitochondria specifically located at mitochondrial inner membranes. The functions of cardiolipin have been primarily related to ATP production^41,42^, curvature stress control and mitochondria cristae morphology^43–45^. Given its function in structuring mitochondrial function and morphology, we suggest that a decrease in cardiolipin levels may partially reflect the loss of mitochondrial cristae and thus dysfunctional mitochondria in the spinal cord of ALS symptomatic rats.

Neurons are known to use lactate from glia to fuel the glycolytic pathway^46,47^. By inducing ROS formation in fruit flies neurons, Liu *et al*. (2017) described lactate being converted to pyruvate and acetyl-CoA, with the latter driving synthesis of fatty acids that are stored as triacylglycerols in lipid droplets^48^. Importantly, these lipid droplets are transported to and accumulate in glial cells for neuroprotection^48,49^. A similar coordination of neurons and astrocytes in the metabolism of fatty acids was recently demonstrated in primary hippocampal neurons and astrocytes from rats^50^. To our knowledge, a detailed lipidomic analysis was not performed by these studies. According to our findings, neutral or storage lipid accumulation was evidenced almost exclusively as a significant increase in cholesteryl esters concentration in spinal cords of ALS symptomatic rats, with no major alterations in the pool of triacylglycerols (Fig. S4C,D). A pertinent question is therefore why cholesteryl esters and not triacylglycerols are stored as neutral lipids in droplets? We hypothesize that the answer relies in part in the efficiency by which lipid droplets can be shuttled from neurons to astrocytes by apolipoproteins (ApoE/D) for neuroprotection as demonstrated by Liu *et al*.^48^.

Accumulation of storage lipids in spinal cords of ALS SOD1-G93A rats was already evidenced by isolation of aberrant astrocytes bearing abundant lipid droplets^19^. Not surprisingly and similar to previous studies with ALS models^21,51,52^, immunohistochemistry of the astrocyte-specific glial fibrillary acidic protein (GFAP) revealed extensive reactive astrogliosis in the spinal cord ventral horn of the SOD1-G93A 120d group (Fig. S6). Nevertheless, the studies evidencing either astrogliosis or aberrant astrocytes in ALS models have not conducted lipidomic analysis. It is thus tempting to suggest that lipids accumulating in aberrant astrocytes^19^ are in fact lipid droplets composed mainly of cholesteryl esters.

Of note, the 6-fold increase of cholesteryl esters in spinal cords of SOD1-G93A 120d group relative to WT 120d group was mostly related to accumulation of molecular species esterified to PUFAs such as arachidonic (20:4), eicosapentaenoic (20:5) and adrenic (22:4) acids. Since mammals cannot synthesize these fatty acids, we speculate that this accumulation likely reflects a protective mechanism against oxidative stress^53^. That is, under elevated ROS, neuronal and glial cells store their highly susceptible membrane-bound PUFAs as cholesteryl esters or triacylglycerols, thereby avoiding membrane lipid peroxidation. It is becoming apparent that sequestering toxic fatty acids into lipid droplets is a widespread cellular strategy to avoid lipotoxicity^48,49,53–55^.

Once protected in lipid droplets, these neutral lipids may be shuttled to glial cells and undergo fatty acid beta-oxidation^46,48,49^. Whether fatty acids can be quantitatively used as fuel for energy metabolism in glial cells remains debatable^56–59^. The controversy is that fatty acid beta-oxidation requires more oxygen than glucose, and may deplete the oxygen demand by neurons^58^. Furthermore, beta-oxidation also generates superoxide anion that may enhance oxidative stress^56,59^, and for this reason neurons likely rely on glial cells for storing excess lipids. We suggest that a delicate balance between neuron-glia lipid transport and energy metabolism (i.e. significant contribution of beta-oxidation) is likely relevant to neuroprotection. In retrospect, any perturbation of this feedback loop may result in augmented neurodegeneration.

**Figure 8:**
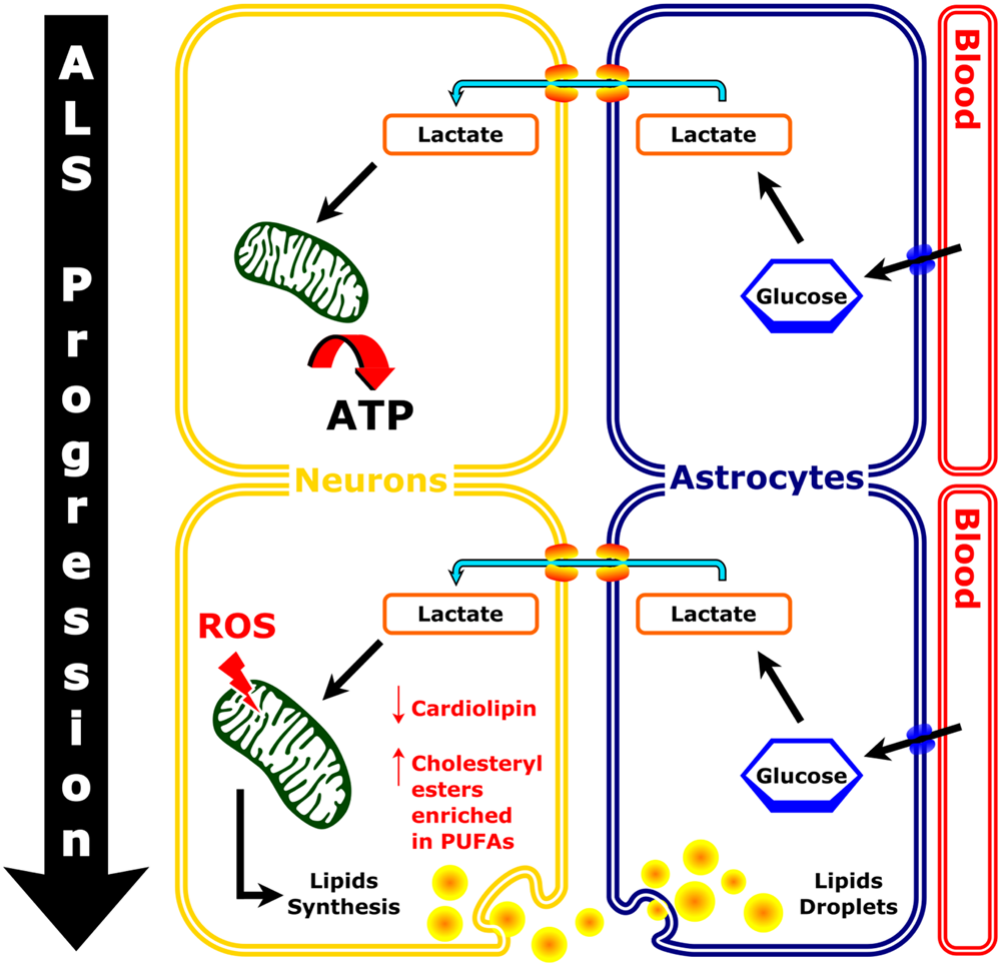
Proposed model for the accumulation of lipid droplets in astrocytes. Under normal conditions, circulating glucose is taken up by astrocytes, converted to lactate and shuttled to neurons for ATP production. Increasing evidences indicate that elevated ROS and mitochondrial defects in neurons trigger lipid droplet formation in astrocytes. In this scenario, our lipidomic data demonstrate that lipid accumulating in the spinal cord are mainly consisted of cholesteryl ester species. Scheme adapted from Liu *et al*., 2017 and Ioannou *et al*.^48,50^.

Here, we attempted to formulate a model shown in Fig. 8 based on: 1) elevated ROS and dysfunctional neuronal mitochondria in ALS (supported by the data showing decreased cardiolipin content in spinal cords of SOD1-G93A 120d group); 2) upregulation of cholesterol synthesis due to altered lactate metabolism in neuronal mitochondria; 3) accumulation of cholesteryl esters composed of PUFAs as a protective mechanism against lipid peroxidation. This hypothetical model is adapted from previously published models^48,50^, with the exception that in our study we performed lipidomics analysis, which revealed significant changes regarding cardiolipin and cholesteryl esters levels related to ALS progression in spinal cord.

Our findings reiterate that alterations in cholesterol metabolism of CNS are associated with ALS^11^. In other neurodegenerative diseases such as Alzheimer’s disease (AD), inhibition of cholesteryl ester synthesis by deletion of ACAT1 prevented Tau phosphorylation and restored cognitive deficits^60^. More recently, the mechanism of cholesteryl esters accumulation avoiding phosphorylated Tau degradation was attributed to a negative regulation of ubiquitin-proteasome system (UPS)^61^. The UPS activity is also down-regulated in ALS^62–64^, leading to accumulation of cytotoxic protein aggregates and ER stress, a hallmark of ALS^8^. In addition, emerging evidence suggests that lipid droplets play a fundamental role in protein aggregation and clearance^54^. Thus, metabolic pathways linked to synthesis and degradation of cholesteryl esters represent potential targets for reduction of ALS disease progression, similar to those reported for AD^61^.

Overall, our study shows the spinal cord is more susceptible to lipid alterations occurring during ALS disease progression than the motor cortex. In addition, our results provide evidence for lipids accumulating as cholesteryl esters in the spinal cords of SOD1-G93A 120d rats, which are under apparent astrogliosis. We proposed a model based on the neuroprotective mechanism against ROS that supports lipid droplets accumulation as cholesteryl esters in astrocytes from the spinal cords of ALS rats. Since fatty acid beta-oxidation is likely limited in the CNS^57,58^, cholesteryl esters might represent a suitable vehicle for lipid transport within and perhaps across the CNS. Thus, cellular processes involving cholesteryl esters metabolism and transport may represent potential targets for treatment of neurodegenerative diseases. The precise analysis of molecular species of lipids as performed in our study may help to further explore the role of oxidative stress regulating lipid metabolism in neurodegenerative diseases and aging.

## Materials and Methods

### Chemicals and reagents

The lipids used as internal standards were 1,2-diheptadecanoyl-sn-glycero-3-phosphocholine (PC 17:0/17:0), 1,2-diheptadecanoyl-sn-glycero-3-phosphoethanolamine (PE 17:0/17:0), 1-heptadecanoyl-2-hydroxy-sn-glycero-3-phosphocoline (LPC 17:0), 1,2-diheptadecanoyl-sn-glycero-3-phosphoserine (PS 17:0/17:0), 1,2-diheptadecanoyl-sn-glycero-3-phospho-(1′-rac-glycerol) (PG 17:0/17:0), 1′, 3′-bis[1,2-dimyristoyl-sn-glycero-3-phospho]-glycerol (CL 14:0 × 4), N-decanoyl-D-erythro-sphingosine (Ceramide d18:1/10:0), 3-O-sulfo-D-galactosyl-ß1-1′-N-heptadecanoyl-D-erythro-sphingosine (Sulfatide d18:1/17:0) and N-heptadecanoyl-D-erytro-sphingosylphosphorylcholine (SM d18:1/17:0), all purchased from Avanti Polar Lipids Inc. (Alabaster, AL. USA). In our analysis, we observed co-elution of internal standards PC (17:0/17:0) and PE (17:0/17:0) with isomers contained in the samples that were composed of 16:0/18:0 fatty acid chain combination. In addition, several other lipid subclasses lacked sufficient amounts or were unavailable (e.g. ubiquinone) in our laboratory to be added as internal standards or were not available for purchase (e.g. 1G-aC, Semino, 1G-AEG). In cases of co-elution and lack of internal standards mentioned above, we used external calibration curves to calculate lipid concentrations. The lipids used for construction of external calibration curves were 1,2-diheptadecanoyl-sn-glycero-3-phosphoinositol (PI 17:0/14:1), free fatty acid (FFA 17:0), (PC 17:0/17:0), (PE 17:0/17:0), d5-1, 3-diheptadecanoyl-glycerol (d5-DAG 17:0 × 2), triacylglycerol (TAG 17:0 × 3), 27-hydroxy-cholesterol and cholesteryl ester (CE 15:0), all obtained from Avanti Polar Lipids Inc (Alabaster, AL. USA). These lipids were all corrected to LPC (17:0) for normalization (see details below). Ammonium formate and ammonium acetate were obtained from Sigma-Aldrich (St Louis, MO, USA) as well as all HPLC grade organic solvents used in this study.

### Animals

Animal study was conducted in accordance with the ethical principles for animal experimentation and conducted according to the guidelines of the National Council for Animal Experimentation Control (Conselho Nacional de Controle de Experimentação Animal – CONCEA, Ministry of Science, Technology, Innovation and Communications, Brazil). Male Sprague Dawley rats overexpressing human SOD1-G93A were obtained from Taconic and bred with wild-type Sprague-Dawley females to establish a colony^65^. Genotyping by PCR to detect exogenous hSOD1 transgene was performed by amplification of ear DNA at 20 days of age. Rats were housed under controlled laboratory conditions, including room temperature, a 12 hours light/12 hours dark cycle with food and water *ad libitum*. Asymptomatic ALS rats (SOD1-G93A 70d; n = 7) and their wild type controls (WT 70d; n = 7) were sacrificed at 73 ± 4 days of age, while symptomatic ALS rats (SOD1-G93A 120d; n = 13) and their control (WT 120d; n = 15) were sacrificed at 122 ± 6 days of age. The criterion for euthanasia of symptomatic SOD1-G93A rats was loss of 15% of their maximum body weight. Rats were fasted for 6 h and anesthetized by isoflurane inhalation at dose of 4% for induction and 2% for maintenance. Motor cortex and spinal cord were collected and stored at −80 °C until further processing. All animal procedures were approved by University of Sao Paulo - Chemistry Institute’s Animal Care and Use Committee under the protocol number 14/2013.

### Lipid extraction

Lipid extraction was performed according to the method established by Yoshida *et al*.^66^. Motor cortex or spinal cord tissues (200 mg) were homogenized in ice by a tissue grinder in 1 mL of 10 mM phosphate buffer (pH 7.4) containing deferoxamine mesylate 100 μM. Briefly, 100 μL of motor cortex or spinal cord homogenate were mixed with 400 μL of phosphate buffer, 400 μL of ice-cold methanol and 100 μL of internal standards (Supplementary Table 1). Next, 1.5 mL of chloroform/ethyl acetate (4:1) was added to the mixture, which was thoroughly vortexed for 30 s. After centrifugation at 1500 g for 2 min at 4 °C, the lower phase containing the total lipid extract (TLE) was transferred to a new tube and dried under N_2_ gas. Simultaneously, yeast samples (10 mg) were extracted and used as quality controls for reproducibility analysis. Dried TLE were redissolved in 100 μL of isopropanol and the injection volume was set at 1 µL. Blanks and quality controls were injected every 5 and 10 samples, respectively.

### Lipidomic analysis

TLEs were analyzed by ESI-Q-TOFMS (Triple TOF^®^ 6600, Sciex, Concord, US) interfaced with an ultra-high performance liquid chromatography (UHPLC Nexera, Shimadzu, Kyoto, Japan). The samples were loaded into a CORTECS^®^ (UPLC^®^ C18 column, 1.6 µm, 2.1 mm i.d. × 100 mm) with a flow rate of 0.2 mL min^−1^ and the oven temperature maintained at 35 °C. For reverse-phase LC, mobile phase A consisted of water/acetonitrile (60:40), while mobile phase B composed of isopropanol/acetonitrile/water (88:10:2). Mobile phases A and B contained ammonium acetate or ammonium formate (at a final concentration of 10 mM) for experiments performed in negative or positive ionization mode, respectively. Lipids were separated by a 20 min linear gradient as follows: from 40 to 100% B over the first 10 min., hold at 100% B from 10–12 min., decreased from 100 to 40% B during 12–13 min., and hold at 40% B from 13–20 min.

The MS was operated in both positive and negative ionization modes, and the scan range set at a mass-to-charge ratio of 200–2000 Da. Data for lipid molecular species identification and quantification was obtained by Information Dependent Acquisition (IDA^®^). Data acquisition using Analyst® 1.7.1 was performed with a cycle time period of 1.05 s with 100 ms acquisition time for MS1 scan and 25 ms acquisition time to obtain the top 36 precursor ions. An ion spray voltage of −4.5 kV and 5.5 kV (for negative and positive modes, respectively) and the cone voltage at +/−80 V were set to analysis. Additional parameters included curtain gas set at 25 psi, nebulizer and heater gases at 45 psi and interface heater of 450 °C.

### Lipidomics data processing

The LC-MS/MS data were analyzed with PeakView^®^. Lipid molecular species were manually identified based on their exact masses, specific fragments and/or neutral losses^67^ and with the help of an in-house manufactured Excel-based macro. Also, a maximum error of 5 mDa was defined for the attribution of the precursor ion. After identification, the area of lipid species was obtained by MS data using MultiQuant^®^. Each peak integration was carefully inspected for correct peak detection and accurate area determination. For quantification, the area ratio of each lipid was calculated by dividing the peak area of the lipid by the corresponding internal standard or using external calibration (Supplementary Table 1). The concentration of lipid species was calculated by either multiplying the area ratio by the concentration of the corresponding internal standard or by external calibration curves relative to LPC (17:0). The external calibration curves used for FFA, PC, PI, PE, cholesterol, CE, DAG and TAG to determine subclass-specific response factors are presented in Supplementary Tables 2 and 3. The total amount of lipids was expressed in µg/g of tissue. Data are presented as mean ± standard error of mean (SEM) (calculated by summing up individual lipid species within each subclass). Note that standards of some lipid subclasses (GalC, 1G-aC, Semino, 1G-AEG and UbQ) were unavailable in the laboratory or commercially and thus lack absolute concentrations. Thus, their concentrations can be compared among samples, but not with other compounds. Data reproducibility analysis was performed by quality controls in both negative and positive ion mode (Supplementary Tables 4 and 5). The peak area and retention time of selected lipids in quality controls (extracted from a yeast sample) were measured at the beginning, after every 10 samples and at the end of the LC-MS/MS experiments.

### Immunofluorescence

Tissue preparation for histological analysis was performed as previously described^68^ with minor modifications. Animals were deeply anesthetized via an intraperitoneal injection of a mixture of ketamine hydrochloride and xylazine and then rapidly perfused transcardially with 0.9% saline, followed by 4% formaldehyde in phosphate-saline buffer, at 4 °C. Spinal cords were removed and postfixed for several days, and then placed in a solution containing 20% sucrose diluted in buffered 4% formaldehyde. The frozen spinal cords (lombar) were mounted on a freezing microtome and cut into 30 μm coronal sections. The slices were collected in cold cryoprotectant solution (0.05 M sodium phosphate buffer, pH 7.3, 30% ethylene glycol, and 20% glycerol) and stored at −20 °C.

The sections were mounted onto adhesive microscope slides and subjected to antigen retrieval by incubation with proteinase K 10 µg/ml at 37 °C for 30 minutes. Following three washes with KPBS, conventional immunofluorescence in fixed tissue was performed as described elsewhere^69^ by incubating washed sections in blocking solution (KPBS containing 1% BSA and 0.4% Triton™ X-100) for 30 min. Using the same buffer solution composition, the sections were incubated overnight at 4 °C with primary antibody anti-glial fibrillary acidic protein (GFAP; Millipore #MAB360). After incubation with the primary antibody, the sections were rinsed in KPBS and incubated with Alexa Fluor® -488 anti-mouse secondary antibodies for 120 min. Sections were then rinsed with KPBS, counterstained with DAPI for nuclear labeling, and coverslip with PVA/DABCO mounting media. Epifluorescence photomicrographs (Nikon^®^ microscope) were processed to enhance contrast and image quality using GIMP (http://www.gimp.org/) and were assembled using Inkscape (http://inkscape.org). Pictures representing different groups received equivalent image treatment.

### Statistical analysis

All statistical analyses were performed with Metaboanalyst (website: www.metaboanalyst.ca). In brief, data were log transformed prior to statistical analyses. All groups were compared by multivariate analysis (sPLS-DA) and one-way ANOVA followed by Tukey’s post-hoc (p < 0.05; FDR-adjusted). We also performed heatmap plots using clusters of lipids that were statistically altered in the different groups (one-way ANOVA p < 0.05; FDR-adjusted) and clusters of samples. For pairwise comparisons of SOD1-G93A 120d group with WT 120d group, we performed multivariate analysis (OPLS-DA) and volcano plots consisting of p-values of t-test (assuming unequal variances; Welch’s test; p < 0.05; FDR-adjusted) and fold change set to >1.5. Graphics were generated using R 3.5.0 base packages (website: www.r-project.org), ggplot2^70^, Metaboanalyst, GraphPad Prism 6 and Excel. Throughout our analysis using Metaboanalyst, we followed the protocols by Xia and others^71^.

## Supplementary information

Supplementary Information

Supplementary table 6. Lipids identified and quantified in Motor Cortex

Supplementary table 7. Lipids identified and quantified in the Spinal Cord

## Data Availability

The datasets generated during and/or analyzed during the current study are available from the corresponding author on reasonable request.
