# Supplementary material for: Alterations in lipid metabolism of spinal cord linked to amyotrophic lateral sclerosis
Source: Sci Rep. 2019 Aug 12;9:11642. doi: 10.1038/s41598-019-48059-7 (PMC6691112; doi:10.1038/s41598-019-48059-7)
Supplement: Supplementary file 1 — Supplementary Information [file 41598_2019_48059_MOESM1_ESM.pdf]

SUPPORTING INFORMATION

Alterations in lipid metabolism of spinal cord linked to amyotrophic lateral sclerosis

Adriano Britto Chaves-Filho[1,*], Isabella Fernanda Dantas Pinto[1,*], Lucas Souza Dantas[1], Andre Machado Xavier[2], Alex Inague[1], Rodrigo Lucas Faria[1], Marisa H. G. Medeiros[1], Isaias Glezer[2], Marcos Yukio Yoshinaga[1], Sayuri Miyamoto[1,¥]

1. Supplementary figures and tables

2. Supplementary references

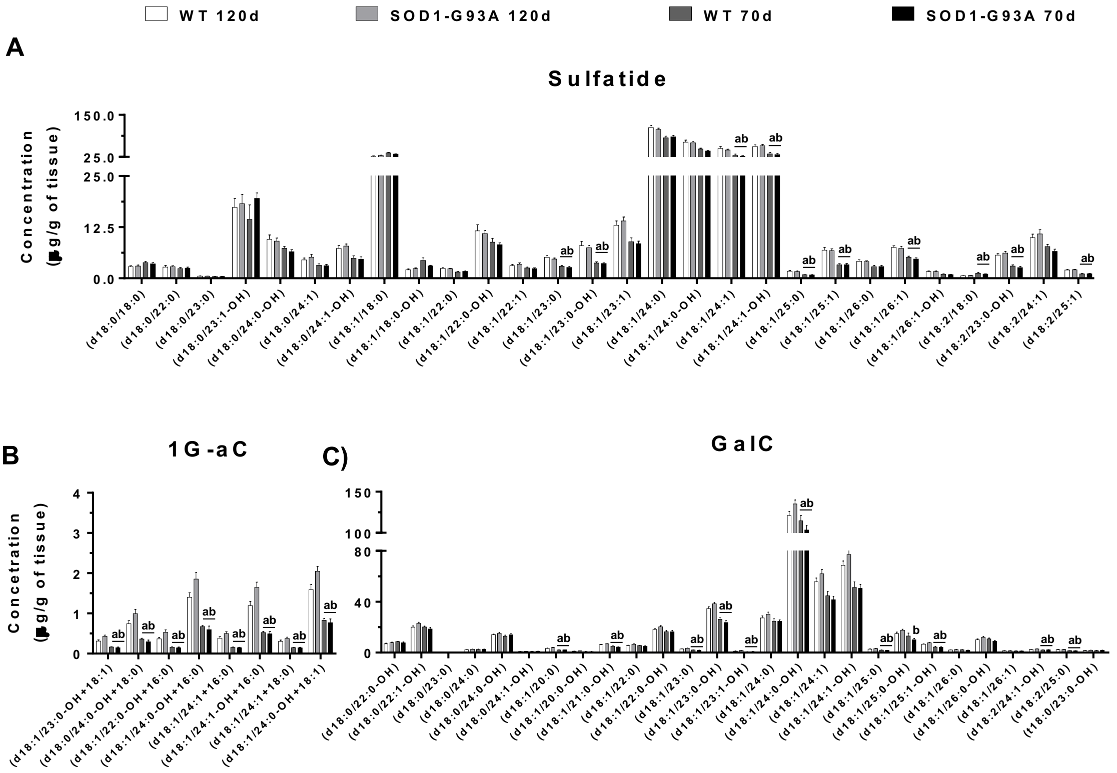

Figure S1. Altered levels of sphingolipid species in motor cortex of SOD1-G93A and WT groups at 70 and 120 days old. Sulfatide (A), monoglycosylated-acylceramide (1G-aC), and galactosylceramide (GalC) molecular species. Data are shown as the mean ± SEM. Statistical significance was evaluated by one-way ANOVA followed by Tukey's post-test using Metaboanalyst ($p < 0.05$; FDR-adjusted). a = different when compared to WT 120d group; b = different when compared to SOD1-G93A 120d group.

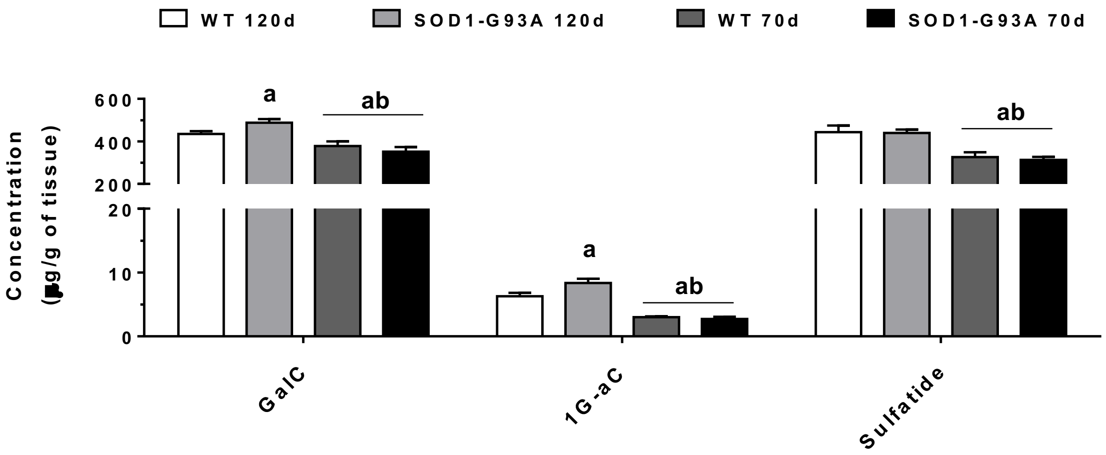

Figure S2. Total levels of the GalC, 1G-aC and sulfatide in motor cortex of SOD1-G93A and WT groups at 70 and 120 days old. Data are shown as the mean ± SEM. Statistical significance was evaluated by one-way ANOVA followed by Tukey's post-test using Metaboanalyst ($p < 0.05$; FDR-adjusted). a = different when compared to WT 120d group; b = different when compared to SOD1-G93A 120d group.

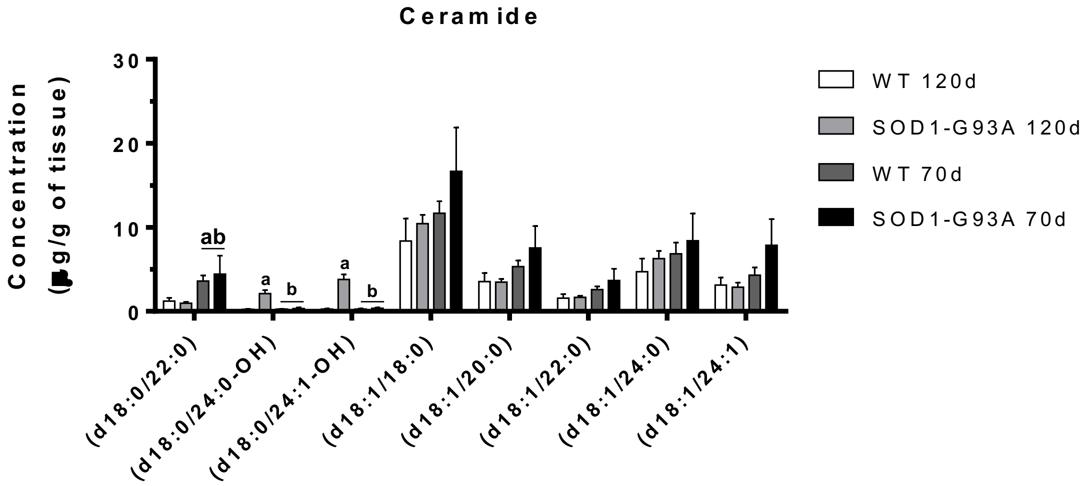

Figure S3. Altered levels of ceramide molecular species in spinal cord of of SOD1-G93A and WT groups at 70 and 120 days old. Data are shown as the mean ± SEM. Statistical significance was evaluated by one-way ANOVA followed by Tukey's post-test using Metaboanalyst ($p < 0.05$; FDR-adjusted). a = different when compared to WT 120d group; b = different when compared to SOD1-G93A 120d group.

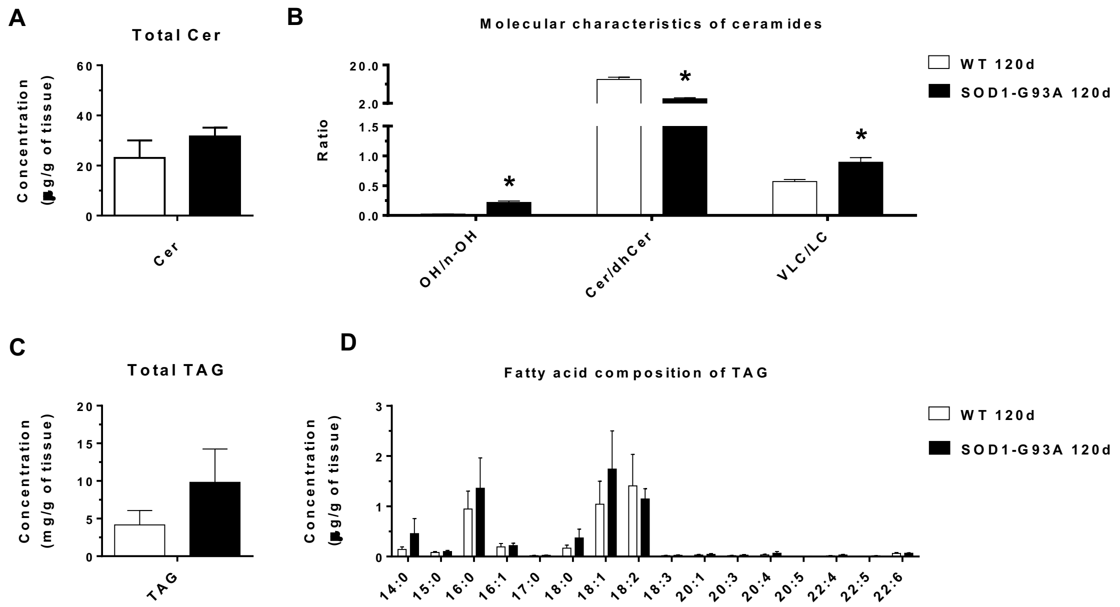

Figure S4. Profile of the altered ceramides (Cer) and triacylglycerols (TAG) in spinal cord of SOD1-G93A 120d and WT 120d groups. (A) Concentration of total ceramides. (B) Molecular characteristics of ceramides. (C) Concentration of total TAGs. (D) Fatty acid composition of TAGs. Data are shown as mean ± SEM. Statistical significance was evaluated by t-test ($p < 0.05$; FDR-adjusted) using Metaboanalyst. (*) Different when compared to the WT 120d group.

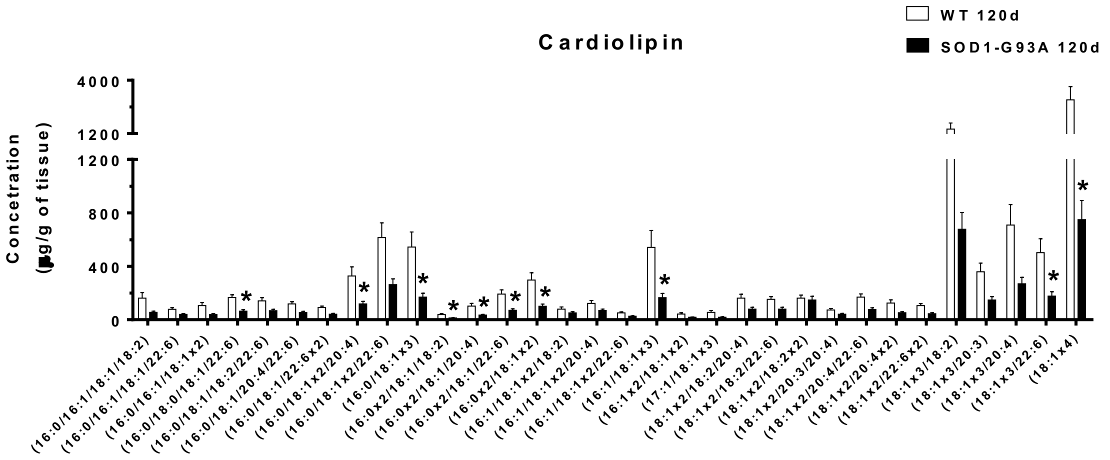

Figure S5. Decreased content of cardiolipin molecular species in spinal cord of SOD1-G93A 120d compared to WT 120d groups. Data are shown as the mean ± SEM. Statistical significance was evaluated by one-way ANOVA followed by Tukey's post-test using Metaboanalyst ($p < 0.05$; FDR-adjusted). (*) Different when compared to WT 120d group.

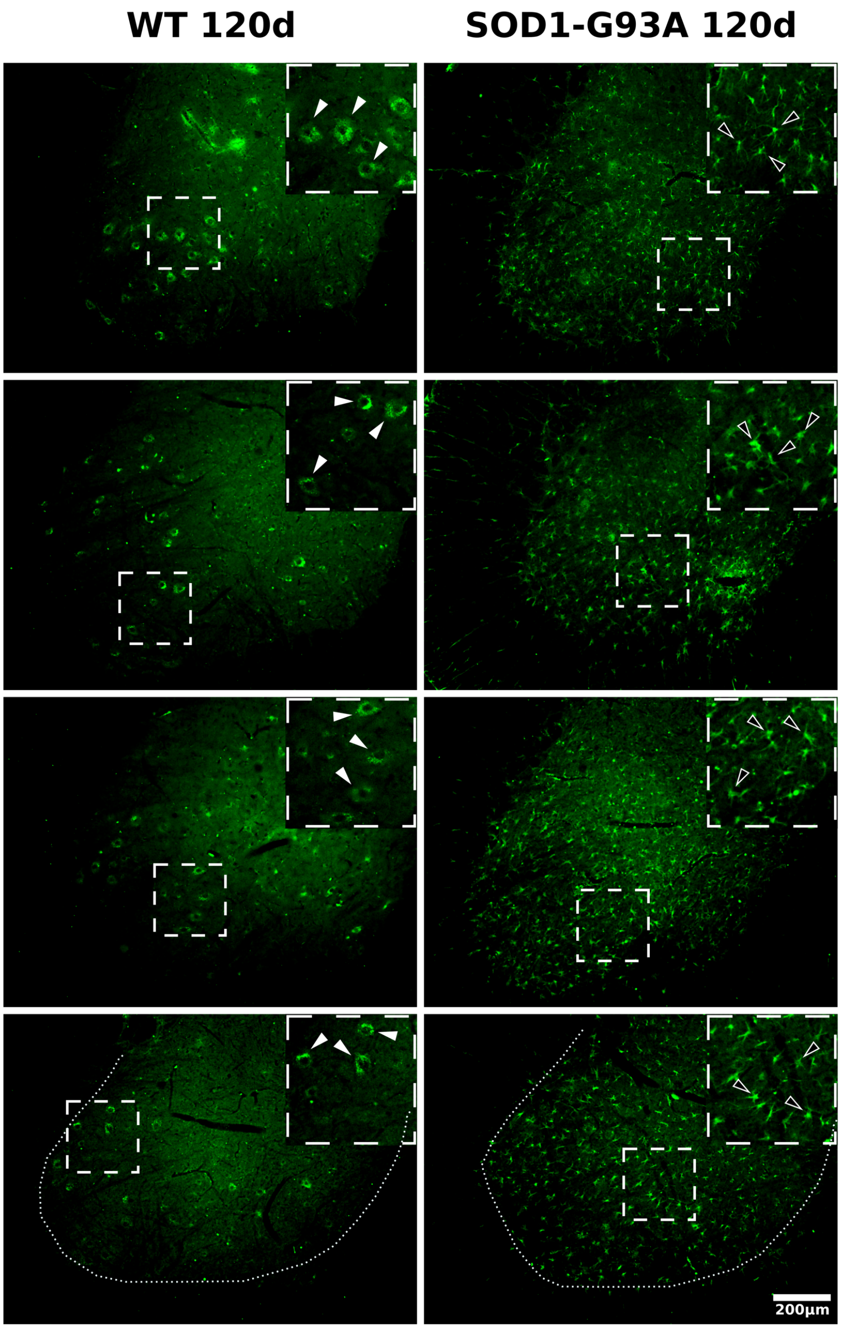

Figure S6. GFAP staining in spinal cord of SOD1-G93A 120d and WT 120d rats. Spinal cord sections were immunostained for GFAP. The ventral gray matter of spinal cords from SOD1 G93A rats present intense astrogliosis as depicted by the microphotographs (each picture is representative of a different animal). Inserts show higher magnification, depicting stained astrocytes (empty arrowheads) in mutant animals. In control animals, large neurons show autofluorescence (full arrowheads), which is typical of age-related lipofuscin accumulation in motor neurons of wild type animals, but absent in SOD1-linked ALS animals[1]. The ventral horn area is surrounded by dashed lines. Scale bars are indicated.

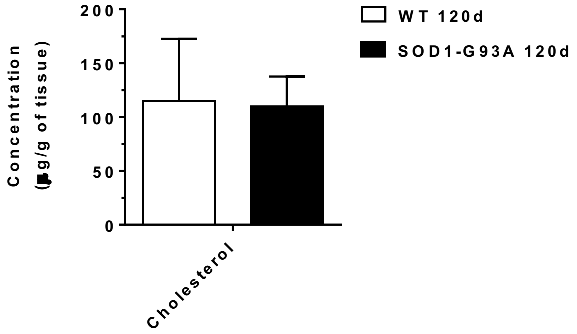

Figure S7. Cholesterol levels in spinal cord of SOD1-G93A 120d and WT 120d groups. Data are shown as the mean ± SEM. Statistical significance was evaluated by t-test ($p < 0.05$; FDR-adjusted) using Metaboanalyst. (*) Different when compared to WT 120d group.

Supplementary table 1. Internal Standard used for the semi quantification.

Internal Standard	Work concentration (ng/µL)	Quantified lipid classes
Cer (d18:1/10:0)	10	Cer, **GalC, **1G-aC
CL (14:0x4)	20	CL
LPC (17:0)	20	*FFA, *PC, *PI, *PE, *Cholesterol, *CE, *DAG, *TAG, **UbQ, **Semino, **1G-AEG
Sulfatide (d18:1/17:0)	20	Sulfatide
PG (17:0/17:0)	20	PG
PS (17:0/17:0)	20	PS, pPS
SM (d18:1/17:0)	16	SM

(*) An external calibration curve relative to LPC (17:0) was used for FFA, PC, PI, PE, cholesterol, CE, DAG and TAG to determine class specific response factors. This calibration was performed using five different concentrations (from ng to pg per injection) of FFA (17:0), PC (17:0/17:0), PI (14:1/17:0), PE (17:0/17:0), 27-hydroxy-cholesterol, CE (15:0), d5-DAG (17:0x2) and TAG (17:0x3). Response factor was calculated by the ratio of the slope of external standards against LPC (17:0). The external calibration yielded a correction factor or response factor of 0.905 (FFA), 1.752 (PC), 0.085 (PE), 0.169 (PI), 0.032 (25-hydroxy-Cholesterol), 0.002 (CE), 0.019 (DAG) and 0.033 (TAG) relative to LPC (17:0). (**) No external calibration was performed for GalC, 1G-aC, Semino, 1G-AEG and UbQ, and so the concentration values are not absolute. The values need to be taken with caution, since they can only be compared within samples and not with other lipid compounds.

Supplementary table 2. External calibration performed in negative ion mode.

Internal standard	slope	r^2	Calibrated range (ng)
LPC (17:0)	288815	0.998	0.02 – 10.00
FFA (17:0)	261446	0.996	0.03 – 1.00
PC (17:0/17:0)	506077	0.998	0.02 – 10.00
PI (17:0/14:1)	48891	0.992	0.03 – 1.00
PE (17:0/17:0)	24635	0.811	0.02 – 10.00

(*) An external calibration curve relative to LPC (17:0) was used for FFA, PC, PI and PE to determine class specific response factors. This calibration was performed using five different concentrations (from ng to pg per injection) of FFA (17:0), PC (17:0/17:0), PI (14:1/17:0) and PE (17:0/17:0). Response factor was calculated by the ratio of the slope of external standards against LPC (17:0). The external calibration yielded a correction factor or response factor of 0.905 (FFA), 1.752 (PC), 0.169 (PI) and 0.085 (PE) relative to LPC (17:0).

Supplementary table 3. External calibration performed in positive ion mode.

Internal standard	slope	r^2	Calibrated range (ng)
LPC (17:0)	1281769	0.999	0.02 – 10.00
27-hydroxy-Ch	40830	0.983	0.02 – 1.00
CE (15:0)	1957	0.985	0.02 – 1.00
d5-DAG (17:0x2)	24140	0.973	0.02 – 1.00
TAG (17:0x3)	41940	0.960	0.02 – 1.00

(*) An external calibration curve relative to LPC (17:0) was used for cholesterol, CE, DAG and TAG to determine class specific response factors. This calibration was performed using five different concentrations (from ng to pg per injection) of 27-hydroxy-cholesterol, CE (15:0), DAG (17:0/17:0) and TAG (17:0x3). Response factor was calculated by the ratio of the slope of external standards against LPC (17:0). The external calibration yielded a correction factor or response factor of 0.032 (25-hydroxy-Cholesterol), 0.002 (CE), 0.019 (DAG) and 0.033 (TAG) relative to LPC (17:0).

Supplementary table 4. Reproducibility of quality controls analyzed in negative ion mode.

Lipid species	Area (x 10^5)	CV (%)	Retention time (min)
Cer (t18:0/26:0)	6.6 ± 0.4	6.4	11.40 ± 0.01
Cer (t18:0/26:0-OH)	5.6 ± 0.3	4.7	11.26 ± 0.00
Cer (t18:0/26:0-OH)	7.0 ± 0.4	5.6	11.26 ± 0.00
FFA (18:1)	7.4 ± 0.4	5.1	5.27 ± 0.02
LysoPC (16:1)	2.9 ± 0.1	3.7	3.11 ± 0.01
LysoPC (17:0)	13.5 ± 0.6	4.6	4.92 ± 0.01
LysoPE (16:1)	3.6 ± 0.1	3.0	3.28 ± 0.01
PC (16:0/18:1)	29.8 ± 1.2	4.2	9.59 ± 0.01
PC (16:1/18:1)	61.7 ± 1.7	2.8	9.11 ± 0.01
PE (16:0/18:1)	10.3 ± 0.3	3.0	9.69 ± 0.01
PE (16:1/18:1)	83.9 ± 2.1	2.6	9.23 ± 0.01
PE (16:1/18:2)	12.1 ± 0.7	5.7	8.90 ± 0.01
PI (16:0/18:1)	4.2 ± 0.5	12.7	8.81 ± 0.01
SM (d18:1/17:0)	10.5 ± 0.3	3.1	9.36 ± 0.01
Sulfatide (d18:1/17:0)	3.1 ± 0.1	4.8	8.46 ± 0.02

Lipid extracts obtained from yeast (n=9) were used as quality control and injected after every 10 samples of motor cortex or spinal cord. Lipid extraction of yeast was performed as described for motor cortex and spinal cord. Area and retention time of lipid species are shown as mean ± S.D.

Supplementary table 5. Reproducibility of quality controls analyzed in positive ion mode.

Lipid species	Area (x 10^5)	CV (%)	Retention time (min)
TAG (16:0/16:1/18:1)	15.6 ± 1.6	10.4	12.26 ± 0.01
TAG (16:0/18:0/18:1)	2.6 ± 0.4	14.5	12.70 ± 0.01
TAG (16:0/18:1x2)	10.1 ± 1.2	11.5	12.47 ± 0.01
TAG (16:1/18:1x2)	20.4 ± 2.0	9.8	12.25 ± 0.01
TAG (16:1x2/18:1)	16.1 ± 1.8	11.2	12.05 ± 0.01
TAG (18:0/18:1x2)	2.3 ± 0.3	12.3	12.69 ± 0.01

Lipid extracts obtained from yeast (n=7) were used as quality control and injected after every 10 samples of motor cortex or spinal cord. Lipid extraction of yeast was performed as described for motor cortex and spinal cord. Area and retention time of lipid species are shown as mean ± S.D.

2. Supplementary references

1. Bandyopadhyay, U., Nagy, M., Fenton, W. A. & Horwich, A. L. Absence of lipofuscin in motor neurons of SOD1-linked ALS mice. *Proc. Natl. Acad. Sci.* **111**, 11055–11060 (2014).
